# Selection procedure human medicine and psychology at the University of Witten/Herdecke: adaptation to the virtual zoom room

**DOI:** 10.3205/zma001363

**Published:** 2020-12-03

**Authors:** Michaela Zupanic, Judith Schulze-Rohr, Julia Nitsche, Thomas Ostermann, Marzellus Hofmann, Jan P. Ehlers

**Affiliations:** 1 Witten/Herdecke University, Faculty of Health, Interprofessional and Collaborative Didactics, Witten, Germany; 2 Witten/Herdecke University, Faculty of Health, Witten, Germany; 3 Witten/Herdecke University, Faculty of Health, Didactics and Educational Research in Health Science, Witten, Germany; 4 Witten/Herdecke University, Faculty of Health, Department of Psychologie and Psychotherapy, Witten, Germany; 5 Witten/Herdecke University, Faculty of Health, Students Dean Office, Witten, Germany

**Keywords:** selection procedure human medicine, selection procedure psychology, virtual selection interviews, online-assessment, acceptance

## Abstract

**Introduction:** For the selection of students for the winter semester 2020/21, the established selection procedure of the University of Witten/Herdecke (UW/H) was adapted to the virtual space in view of the current contact ban and recommended keeping of distance. The three stations in the second step of the procedure, the biographical one-on-one interview, presentation and discussion on a subject-specific topic as well as multiple mini interviews (MMI) on the social skills of the applicants were audiovisual and in real time in zoom meetings.

**Project description: **The medical, psychological and student reviewers were prepared for the virtual selection procedure in training sessions. Three weeks before the selection days, the applicants received information on the technical requirements for the interviews and on data protection for the persons affected by the collection of personal data. The evaluation of the virtual selection procedure was carried out by the reviewers using an online questionnaire with 8 socio-demographic, 5 organizational, 8 content and 3 open questions.

**Results: **The 108 reviewers conducted selection interviews in tandems (medical/psychological and student reviewers) with 178 applicants for human medicine and 105 applicants for psychology. The online evaluation by 58 reviewers (response rate 53.7%) showed a positive agreement with the virtual selection procedure, with a more favorable assessment of organization and content by the medical and psychological reviewers compared to the student reviewers.

**Discussion: **The adequate adaptation of the selection procedure of the UW/H to the virtual zoom room as well as its acceptance are confirmed by the successful execution of the selection days for the students for the winter semester 2020/21 and the evaluation of the reviewers.

**Conclusion:** The results and analysis of this exceptional situation will be used to also conduct the upcoming selection procedure for the summer semester 2021 in the virtual space. A valid assessment for the future use of a virtual selection procedure as a possible supplement to the personal selection interviews at the University of Witten/Herdecke remains to be made.

## Zusammenfassung

**Einleitung:** Zur Auswahl der Studierenden zum Wintersemester 2020/21 wurde in Anbetracht der geltenden Kontaktsperre und empfohlenen Abstandswahrung das etablierte Auswahlverfahren der Universität Witten/Herdecke (UW/H) an den virtuellen Raum adaptiert. Die drei Stationen im zweiten Schritt des Verfahrens, das biografische Einzelgespräch, Referat und Diskussion zu einem fachspezifischen Thema sowie Multiple Mini Interviews (MMI) zu den sozialen Kompetenzen der Bewerbenden erfolgten audiovisuell und in Echtzeit in Zoom-Meetings.

**Projektbeschreibung:** Die ärztlichen, psychologischen und studentischen Gutachtenden wurden in Schulungen auf das virtuelle Auswahlverfahren vorbereitet. Die Bewerbenden erhielten drei Wochen vor den Auswahltagen Informationen zu den technischen Voraussetzungen für die Gespräche und für die von der Erhebung personenbezogener Daten betroffenen Personen bzgl. Datenschutz. Die Evaluation des virtuellen Auswahlverfahrens erfolgte durch die Gutachtenden mit einem Online-Fragebogen mit 8 soziodemografischen, 5 organisatorischen, 8 inhaltlichen und 3 offenen Fragen.

**Ergebnisse: **Die 108 Gutachtenden führten in Tandems (ärztliche/psychologische und studentische Gutachter*innen) Auswahlgespräche mit 178 Bewerbenden für Humanmedizin und 105 Bewerbenden für Psychologie. Die Online-Evaluation durch 58 Gutachtende (Rücklaufquote 53,7%) ergab eine positive Zustimmung zum virtuellen Auswahlverfahren, mit günstigerer Bewertung von Organisation und Inhalt durch die ärztlichen und psychologischen Gutachtenden im Vergleich zu den studentischen Gutachtenden.

**Diskussion: **Die adäquate Adaptation des Auswahlverfahrens der UW/H an den virtuellen Zoom-Raum sowie dessen Akzeptanz werden durch die erfolgreiche Durchführung der Auswahltage für die Studierenden zum Wintersemester 2020/21 und die Evaluation der Gutachtenden bestätigt.

**Schlussfolgerung: **Die Ergebnisse und Analyse dieser Ausnahmesituation werden genutzt, um auch das kommende Auswahlverfahren zum Sommersemester 2021 im virtuellen Raum durchzuführen. Offen bleibt noch eine valide Einschätzung für die zukünftige Nutzung eines virtuellen Auswahlverfahrens als mögliche Ergänzung zu den persönlichen Auswahlgesprächen an der Universität Witten/Herdecke.

## Introduction

The international state of research on selection procedures for medical students is also taken into account at German medical faculties in the use of cognitive and psychosocial abilities as criteria for predicting the success of studies [1]. At the University of Witten/Herdecke (UW/H), student selection is a two-step process with a letter of motivation in the first step and a selection day with three different interview situations in the second [2]. In view of the Sars-CoV-2 pandemic, it was necessary to create an alternative for the selection process of medical and psychology students for the winter semester 2020/21. The UW/H decided on a contact-free version of the selection days and their relocation to virtual rooms of the remote conference service Zoom [https://www.zoom.us/], which, among other things, enables audiovisual and real-time communication with several participants. The possibility of conducting job interviews virtually is a relatively new discipline, although this format offers applicants more flexibility at lower costs and is an acceptable alternative to personal interviews for the faculty [3]. 

This project report describes the adaptation of the procedure to virtual space and its acceptance by the reviewers of the UW/H. 

## Project description

### Adaptation of the process

For reasons of fairness and practicability, the virtual selection procedure was implemented in zoom meetings with the following three stations of 30 minutes each:

1. biographical individual interview with two reviewers in tandem (medical/psychological and student reviewer);
2. presentation on a topic of your own choice and subsequent discussion with the two reviewers;
3. two different tasks in a multiple mini interview.

#### Training of the reviewers

For the medical and psychological reviewers (n=53), online training courses on the functionality and handling of the virtual zoom rooms were held in preparation. For the student reviewers (n=55) participating for the first time, the focus was on explaining the selection criteria and objectives at the various stations.

#### Information on the selection days

With the invitation three weeks before their respective selection day, the applicants received information for the persons affected by the collection of personal data from the data protection officer of UW/H. Three days before, they received their number and the individual schedule with information on when they are expected to arrive in which zoom room for which station in the selection process. On the selection day, the reviewers remained in their tandem in the same zoom room and were able to assess the applicants during their rotation period. The logistics of a selection day (morning) are shown in table 1 (Tab. 1).

In order to examine the feasibility and acceptance of the virtual selection procedure, the reviewers were sent an online questionnaire after the selection days (ethics vote of the UW/H 88/2020). The questionnaire contains 8 socio-demographic questions (e.g. age, gender, activity as an reviewer) and on a five-level Likert scale (1=strongly disagree ... 5=strongly agree) 5 organizational questions (e.g. procedure, technology) and 8 content questions (e.g. fairness, contact, assessment of the applicants) as well as 3 final open questions (see attachment 1 ). 

## Results

### Selection Days

In the summer semester 2020, six selection days were held for the model course of study in human medicine. A total of 83 reviewers (41 medical, 42 student reviewers) examined 178 applicants in the selection interviews for their motivation and suitability for UW/H. The selection for the study programs in the Department of Psychology and Psychotherapy took place over four days. A total of 25 reviewers (12 psychological, 13 student reviewers) examined 90 applicants for the Bachelor's program in Psychology and 15 applicants for the Master's program in Clinical Psychology. 

#### Evaluation

**Figure 1:**
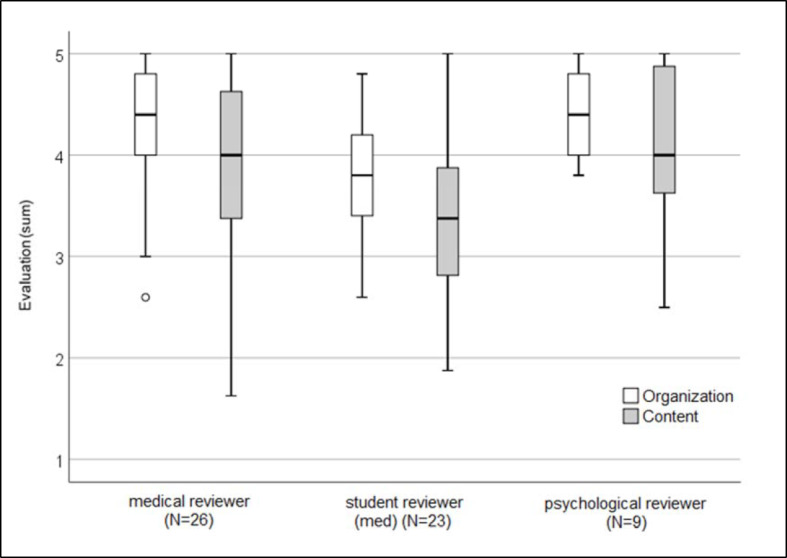
Evaluation of the virtual selection procedure with respect to organization and content by the reviewers (n=58) (box plot)

58 reviewers (21 female, 37 male) participated in the online evaluation. The average sum of the evaluation of 3.9±0.7 (range 1-5) corresponds to an approval of the virtual selection procedure, in which 89.7% (n=52) of the reviewers would participate again. Student reviewers assessed the virtual selection procedure significantly more critically than medical and psychological reviewers with respect to organization (Kruskal-Wallis-H=11.6, p=.003) and content (Kruskal-Wallis-H=7.1, p=.028), as shown in figure 1 (Fig. 1).

## Discussion

The decision for the adaptation of the selection procedure of the UW/H to the virtual zoom room can be evaluated as adequate, since all selection days could be carried out successfully. The evaluation by the reviewers confirms the acceptance of the virtual selection procedure, although the student reviewers judge this change more critically, as already the introduction of the MMIs into the selection procedure [4].

However, the benefit of digital methods should be considered in terms of whether the established techniques of personnel selection can be surpassed [5]. This means that the virtual selection process should yield comparable results to the university's own selection process, which has been in place for more than 30 years [6] and in which great value is placed on the "personality and talent" of applicants, particularly in the Departments of Human Medicine and Psychology of the Faculty of Health. 

## Conclusion

The results and analysis of this exceptional situation will be used to conduct the upcoming selection procedure for the summer semester 2021 in virtual space. A valid assessment for the future use of a virtual selection procedure as a possible supplement to the personal selection interviews at the University of Witten/Herdecke remains to be made.

## Acknowledgements

The authors would like to thank the staff of the Admissions Offices for Human Medicine and Psychology, Cornelia Bock, Elke Moch, Dagmar Reinders and Petra Stammnitz as well as the medical students Marie Dahlke and Konstantin März for their active support in adapting the selection procedure to the virtual space.

## Competing interests

The authors declare that they have no competing interests. 

## Supplementary Material

Evaluation of a virtual selection procedure (VSP) for human medicine and psychology at the University of Witten/Herdecke

## Figures and Tables

**Table 1:**
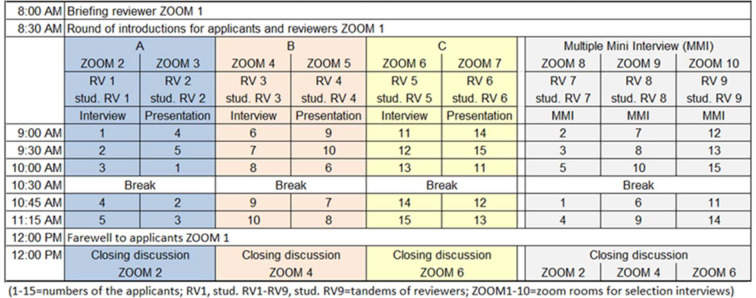
Schedule for a morning in the virtual selection procedure (15 applicants, 9 tandems with reviewers, 10 zoom rooms)
