# Supplementary material for: Selection procedure human medicine and psychology at the University of Witten/Herdecke: adaptation to the virtual zoom room
Source: GMS J Med Educ. 2020 Dec 3;37(7):Doc70. doi: 10.3205/zma001363 (PMC7740028; doi:10.3205/zma001363)
Supplement: Evaluation of a virtual selection procedure (VSP) for human medicine and psychology at the University of Witten/Herdecke [file JME-37-70-s-001.pdf]

ATTACHMENT 1: QUESTIONNAIRE FOR THE ONLINE SURVEY

EVALUATION OF A VIRTUAL SELECTION PROCEDURE (VSP) FOR HUMAN MEDICINE AND PSYCHOLOGY AT THE UNIVERSITY OF WITTEN / HERDECKE

DEAR REVIEWERS,

THANK YOU VERY MUCH FOR YOUR PARTICIPATION IN THE VIRTUAL SELECTION PROCEDURE OF UW / H AND FOR ANSWERING THIS QUESTIONNAIRE. WITH THE DECISION TO PARTICIPATE IN THIS SHORT QUESTIONNAIRE, YOU ARE SUPPORTING THE DEVELOPMENT OF NEW KNOWLEDGE CONCERNING THE SUITABILITY OF A VIRTUAL SELECTION PROCEDURE AT UW / H.
THIS PARTICIPATION IS VOLUNTARY.

IN ORDER TO ASSESS THE SUITABILITY, THIS ONLINE QUESTIONNAIRE WAS CREATED AS PART OF A DOCTORAL THESIS. THE QUESTIONS ARE TREATED COMPLETELY ANONYMOUSLY, SO NONE OF THE STATEMENTS CAN BE TRACED BACK TO YOUR PERSON. FURTHERMORE, THE RESULTS WILL BE USED ONLY FOR THE SCIENTIFIC PURPOSE OF THE DOCTORAL THESIS.

PARTICIPATION DURATION: APPROX. 10 MINS

PERIOD FOR ANSWERING THE QUESTIONNAIRE: JUNE 30TH - JULY 31TH 2020

BY PARTICIPATING IN THIS ONLINE SURVEY, YOU AGREE TO THE ANONYMOUS PROCESSING OF THE PROVIDED DATA.
❑ **I agree and would like to take part in the survey.**

I, JUDITH SCHULZE-ROHR, STUDENT OF HUMAN MEDICINE, WILL BE AVAILABLE FOR ANY QUESTIONS AND FOR INFORMATION (JUDITH.SCHULZE-ROHR@UNI-WH.DE).

Attachment 1 to: Zupanic M, Schulze-Rohr J, Nitsche J, Ostermann T, Hofmann M, Ehlers JP. *Selection procedure human medicine and psychology at the University of Witten/Herdecke: adaptation tot he virtual zoomroom.* GMS J Med Educ. 2020;37(7):Doc70. DOI: 10.3205/zma001363

QUESTIONS ABOUT THE PERSON AND ABOUT THE ACTIVITY AS REVIEWER

1. AGE: ___ YEARS

2. SEX: □ FEMALE □ MALE □ DIVERSE

3. HOW OFTEN HAVE YOU ALREADY PARTICIPATED AS REVIEWER IN SELECTION DAYS OF THE UW / H?
   □ NEVER □ 1 – 5 TIMES □ 6 – 10 TIMES □ 11 – 20 TIMES □ > 20 TIMES

4. HOW OFTEN HAVE YOU ALREADY BEEN A REVIEWER IN A VIRTUAL SELECTION PROCEDURE (OUTSIDE THE UW / H)?

   _________ TIMES

5. ON WHICH VIRTUAL SELECTION DAYS DID YOU PARTICIPATE AS A REVIEWER IN THE SUMMER SEMESTER 2020?

   □ MAY 9TH – HUMAN MEDICINE
   □ MAY 10TH – HUMAN MEDICINE
   □ JUNE 6TH – HUMAN MEDICINE
   □ JUNE 7TH – HUMAN MEDICINE
   □ JUNE 20TH – HUMAN MEDICINE
   □ JUNE 21ST – HUMAN MEDICINE
   □ MAY 14TH – PSYCHOLOGY (B.SC.)
   □ JUNE 4TH – PSYCHOLOGY (B. SC.)
   □ JUNE 25TH – PSYCHOLOGY (B.SC.)

6. TO WHICH FACULTY AND DEPARTMENT DO YOU BELONG?

   □ FACULTY OF HEALTH
      □ DEPARTMENT OF HUMAN MEDICINE
      □ DEPARTMENT OF NURSING SCIENCE
      □ DEPARTMENT OF PSYCHOLOGY
      □ DEPARTMENT OF DENTISTRY
   □ FACULTY OF ECONOMICS
   □ FACULTY OF CULTURAL REFLECTION

7. WHAT WAS YOUR PART IN THE TANDEM?

   □ STUDENT REVIEWER
   □ MEDICAL REVIEWER
   □ PSYCHOLOGICAL REVIEWER
   □ OTHER REVIEWER: ____

Attachment 1 to: Zupanic M, Schulze-Rohr J, Nitsche J, Ostermann T, Hofmann M, Ehlers JP. *Selection procedure human medicine and psychology at the University of Witten/Herdecke: adaptation tot he virtual zoomroom.* GMS J Med Educ. 2020;37(7):Doc70. DOI: 10.3205/zma001363

8. WHICH STATION(S) WERE YOU PART OF AS REVIEWER?

- ☐ BIOGRAPHICAL INTERVIEW
- ☐ INDIVIDUAL TALK WITH PRESENTATION AND DISCUSSION
- ☐ MULTIPLE MINI INTERVIEWS (MMI)

QUESTIONS ABOUT THE VIRTUAL SELECTION PROCEDURE (VSP)

ORGANIZATIONAL

9. I WAS SATISFIED WITH THE INFORMATION AND THE PREPARATION FOR THE VSP.

strongly agree	agree	neither agree nor disagree	disagree	strongly disagree
☐	☐	☐	☐	☐

10. I DIDN'T NEED TECHNICAL PREPARATION FOR THE VSP.

strongly agree	agree	neither agree nor disagree	disagree	strongly disagree
☐	☐	☐	☐	☐

11. THE ADAPTATION OF THE STATIONS IN THE SELECTION PROCEDURE TO THE VIRTUAL ROOM SEEMED REASONABLE.

strongly agree	agree	neither agree nor disagree	disagree	strongly disagree
☐	☐	☐	☐	☐

☐ I WAS A REVIEWER FOR THE FIRST TIME.

12. THE ORGANIZATIONAL PROCEDURE OF THE SELECTION DAY WAS SMOOTH.

strongly agree	agree	neither agree nor disagree	disagree	strongly disagree
☐	☐	☐	☐	☐

13. NO TECHNICAL DIFFICULTIES (AUDIO / VIDEO) OCCURED.

strongly agree	agree	neither agree nor disagree	disagree	strongly disagree
☐	☐	☐	☐	☐

Attachment 1 to: Zupanic M, Schulze-Rohr J, Nitsche J, Ostermann T, Hofmann M, Ehlers JP. *Selection procedure human medicine and psychology at the University of Witten/Herdecke: adaptation tot he virtual zoomroom.* GMS J Med Educ. 2020;37(7):Doc70. DOI: 10.3205/zma001363

CONTENT:

14. I CONSIDER THE VSP AN ADEQUATE OPPORTUNITY TO GET AN APPROPRIATE IMAGE OF THE APPLICANT.

strongly agree	agree	neither agree nor disagree	disagree	strongly disagree
☐	☐	☐	☐	☐

15. I WAS ABLE TO RATE THE APPLICANTS AT MY STATION EASILY AND AWARD THEM THE APPROPRIATE POINTS.

strongly agree	agree	neither agree nor disagree	disagree	strongly disagree
☐	☐	☐	☐	☐

16. THE VSP IS A FAIR AND APPROPRIATE OPPORTUNITY FOR APPLICANTS TO PRESENT THEMSELVES TO THE REVIEWER.

strongly agree	agree	neither agree nor disagree	disagree	strongly disagree
☐	☐	☐	☐	☐

17. MY ABILITY TO EVALUATE CANDIDATES HAS NOT BEEN CHANGED BY THE VSP.

strongly agree	agree	neither agree nor disagree	disagree	strongly disagree
☐	☐	☐	☐	☐

18. THE LACK OF POSSIBILITY OF DIRECT CONTACT HAS IMPEDED THE APPRAISAL OF THE APPLICANTS.

strongly agree	agree	neither agree nor disagree	disagree	strongly disagree
☐	☐	☐	☐	☐

19. IT WAS HARD TO STAY CONCENTRATED THROUGHOUT THE VSP.

strongly agree	agree	neither agree nor disagree	disagree	strongly disagree
☐	☐	☐	☐	☐

20. THE REDUCED OPPORTUNITY TO INTERACT WITH MY TANDEM REVIEWER BOTHERED ME.

strongly agree	agree	neither agree nor disagree	disagree	strongly disagree
☐	☐	☐	☐	☐

21. I WOULD TAKE PART AS A REVIEWER IN A VSP AGAIN.

strongly agree	agree	neither agree nor disagree	disagree	strongly disagree
☐	☐	☐	☐	☐

OPEN QUESTIONS:

1. WHAT ADVANTAGES DO YOU SEE IN CARRYING OUT A VIRTUAL SELECTION PROCEDURE?

   ____
   ____
   ____
   ____
   ____

2. WHAT DISADVANTAGES IN THE VSP DID OCCUR TO YOU?

   ____
   ____
   ____
   ____
   ____

3. ARE THERE ANY OTHER SUGGESTIONS / THOUGHTS ABOUT THE VSP THAT YOU WOULD LIKE TO SHARE WITH US?

   ____
   ____
   ____
   ____
   ____

THANK YOU FOR TAKING PART IN THIS STUDY!

Attachment 1 to: Zupanic M, Schulze-Rohr J, Nitsche J, Ostermann T, Hofmann M, Ehlers JP. *Selection procedure human medicine and psychology at the University of Witten/Herdecke: adaptation tot he virtual zoomroom.* GMS J Med Educ. 2020;37(7):Doc70. DOI: 10.3205/zma001363
